# Long non-coding RNA HOTTIP promotes BCL-2 expression and induces chemoresistance in small cell lung cancer by sponging miR-216a

**DOI:** 10.1038/s41419-017-0113-5

**Published:** 2018-01-24

**Authors:** Yanqin Sun, Bingshuang Hu, Qiongyao Wang, Minting Ye, Qianqian Qiu, Yuanyuan Zhou, Fanrui Zeng, Xiaomin Zhang, Ying Guo, Linlang Guo

**Affiliations:** 1 Department of Pathology, Zhujiang Hospital, Southern Medical University, 253 Gongye Road, Guangzhou, 510282 China; 2 Department of Pathology, Guangdong Medical University, Dongguan, China; 3 Department of Radiotherapy, Zhongshan People’s Hospital, Zhongshan, China; 4 Department of Oncology, Zhujiang Hospital, Southern Medical University, Guangzhou, China; 5 Department of Pathology, Shunde Hospital, Southern Medical University, Foshan, China; 6 Department of Dermatology and STD, Changping Hospital, Dongguan, China; 7 Department of Organ Transplantation, Zhujiang Hospital, Southern Medical University, 253 Gongye Road, 510282 Guangzhou, China

## Abstract

Despite progress in treatment of small cell lung cancer (SCLC), its multidrug chemoresistance and poor prognosis still remain. Recently, we globally assessed long non-coding RNAs (lncRNAs) for contributions to SCLC chemoresistance using microarray data, in vitro and in vivo assays. Here we reported that HOTTIP, encoding a lncRNA that is frequently amplified in SCLC, was associated with SCLC cell chemosensitivity, proliferation, and poor prognosis of SCLC patients. Moreover, mechanistic investigations showed that HOTTIP functioned as an oncogene in SCLC progression by binding miR-216a and abrogating its tumor-suppressive function in this setting. On the other hand, HOTTIP increased the expression of anti-apoptotic factor BCL-2, another important target gene of miR-216a, and jointly enhanced chemoresistance of SCLC by regulating BCL-2. Taken together, our study established a role for HOTTIP in SCLC progression and chemoresistance suggest its candidacy as a new diagnostic and prognostic biomarker for clinical management of SCLC.

## Introduction

As one of the most malignant tumors in the world, lung cancer poses a great threat to people’s health and life. Small cell lung cancer (SCLC) accounts for approximately 15% of all lung cancers at present. The failure of clinical SCLC treatment is mostly due to its rapid growth and rapid development of multidrug resistance (MDR) to chemotherapy. Etoposide (VP-16) or irinotecan plus cisplatin (CDDP) combination therapy is the current front-line standard chemotherapy regimen for SCLC, but MDR occurs shortly after the first successful treatment^1^. Therefore, chemoresistance has become one of the major problems to cause poor prognosis of SCLC.

The transcribed RNAs, which have been classified as non-coding RNAs (ncRNAs), account for >90% of the transcriptome without protein-coding potential^2^. Therein, microRNAs (miRNAs; 18–200 nucleotides) have been extensively studied, thousands of which regulate up to 30% of their protein-encoding target genes^3^. Additionally, long ncRNAs (lncRNAs) whose length over 200 nucleotides, have been identified to play crucial regulatory roles in tissue differentiation, proliferation, migration, invasion, apoptosis, and epigenetic silencing including lncRNA–miRNA interaction and lncRNA–protein interaction^4^. LncRNAs also could act as a competing endogenous RNA or sponge miRNAs to regulate the expression of target mRNAs^5^. Therefore, several lncRNAs have been confirmed as having a critical role in regulating gene expression in tumorigenesis, and its aberrant expression has been functionally associated with many cancers, including lung cancer^5–15^.

The lncRNA HOTTIP (HOXA transcript at the distal tip), a newly identified lncRNA, located at the 5′ end of the HOXA cluster, which is a key locus control element of HOXA genes and distal identity, and is brought into close proximity to the 5′ HOXA genes by chromosomal looping^2^. There is considerable evidence that HOTTIP was recently functionally characterized to play important roles in the differentiation, proliferation, and genome maintenance of various types of human cancers^6,7,10,13,14,16–18^. For instance, Quagliata et al.^13^ proposed a functional role for HOTTIP in the disease progression and predicts outcome in hepatocellular carcinoma. However, the underlying role of HOTTIP in MDR, particularly in SCLC chemoresistance, remains unclearly known.

The aim of this study was to identify the function of lncRNA HOTTIP in SCLC, and to uncover the potential mechanisms by which HOTTIP contributes to SCLC pathogenesis and chemoresistance. We also performed the present study to investigate whether miR-216a mediated this process. Our findings will provide new insights into the molecular functions of lncRNA HOTTIP, as well as its regulatory mechanisms in SCLC tumorigenesis and chemoresistance.

## Results

### HOTTIP expression is increased in human SCLC chemoresistant cell lines and formalin-fixed, paraffin-embedded (FFPE) tissues

**Fig. 1:**
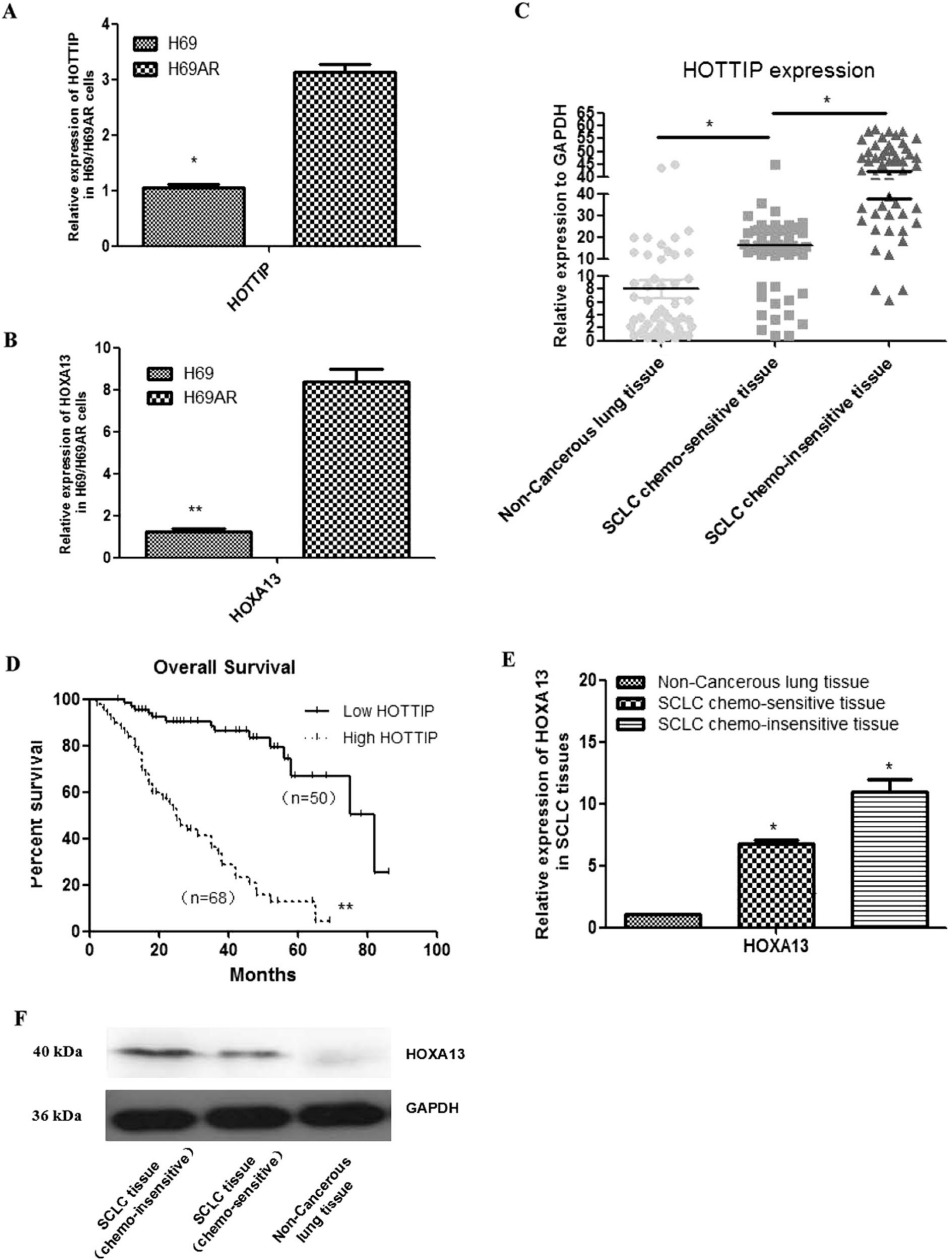
Relative expression of HOTTIP and HOXA13 in cell lines and tissues, as well as clinical significance of HOTTIP in SCLC tissues **a** Expression of HOTTIP in H69 and H69AR cell lines. **b** Expression of HOXA13 in H69 and H69AR cell lines. **c** Expression of HOTTIP in SCLC chemoinsensitive tissues, chemosensitive tissues, and non-cancerous lung tissues counterparts is descending in turn. **d** Patients with high HOTTIP expression had a significantly poorer prognosis than those with low HOTTIP expression. **e** Expression of HOXA13 in SCLC chemoinsensitive tissues, chemosensitive tissues, and non-cancerous lung tissues is descending in turn. **f** Expression of HOXA13 protein is validated in SCLC FFPE tissues by western blot. *P < 0.05; **P < 0.001

To identify lncRNAs overexpressed in SCLC, we performed gene expression array analysis on H69 and H69AR cell lines (Supplementary Figures 1A-1B). One thousand four hundred forty-three lncRNAs statistically significant were involved, their functions are involved in apoptosis, enzyme activity regulation, cell cycle gene regulation, metabolism, signal transduction activity, and so on (Supplementary Table 1). Among them, 16 HOX family members including lncRNA HOTTIP and HOXA13 gene were upregulated >10-fold changes in H69AR compared with H69 cell line (Supplementary Figure 1C), then expression of HOTTIP and HOXA13 were validated in H69 and H69AR cell line by quantitative reverse transcription-PCR (RT-qPCR; Figs. 1a, b).

To further verify these results, the expression of HOTTIP was determined in SCLC tissues and non-cancerous lung tissues by RT-qPCR (Supplementary Figure 1D). HOTTIP expression was significantly upregulated in SCLC chemosensitive tissues compared with non-cancerous lung tissues, whereas HOTTIP expression was significantly upregulated in SCLC chemoinsensitive tissues compared with chemosensitive tissues counterparts (Fig. 1c). Moreover, patients with high HOTTIP expression had a significantly poorer prognosis than those with low HOTTIP expression (Fig. 1d). Expression of HOXA13 is also validated in SCLC FFPE tissues above by western blot (Figs. 1e–f). Examination of the correlation between HOTTIP expression and clinical pathological features showed that HOTTIP upregulation was correlated with disease stage, chemotherapy response, and median survival, but HOTTIP expression was not associated with patient age or gender (Table 1). These results imply that HOTTIP increased expression may be useful in the development of novel prognostic or progression markers and chemoresistance progression for SCLC.

**Table 1:** Association of HOTTIP with clinical parameters

Patients characteristics	HOTTIP expression			
	−	+	*χ* ^2^	*P*-value*
All cases (*N*=115)	27	88		
Age			0.5957	0.4402
≦56	10	40		
>56	17	48		
Gender			0.4183	0.5178
Male	15	55		
Female	12	33		
Disease stage			9.6084	0.0019
Limited disease (LD)	20	33		
Extensive-stage disease (ED)	7	50		
Chemotherapy response			9.6976	0.0018
Chemosensitive	18	35		
Chemoinsensitive	9	69		
Median survival (5–36 months)			23.5898	<0.001
Survival	19	18		
Death	8	70		

### Manipulation of HOTTIP levels in SCLC cell lines

**Fig. 2:**
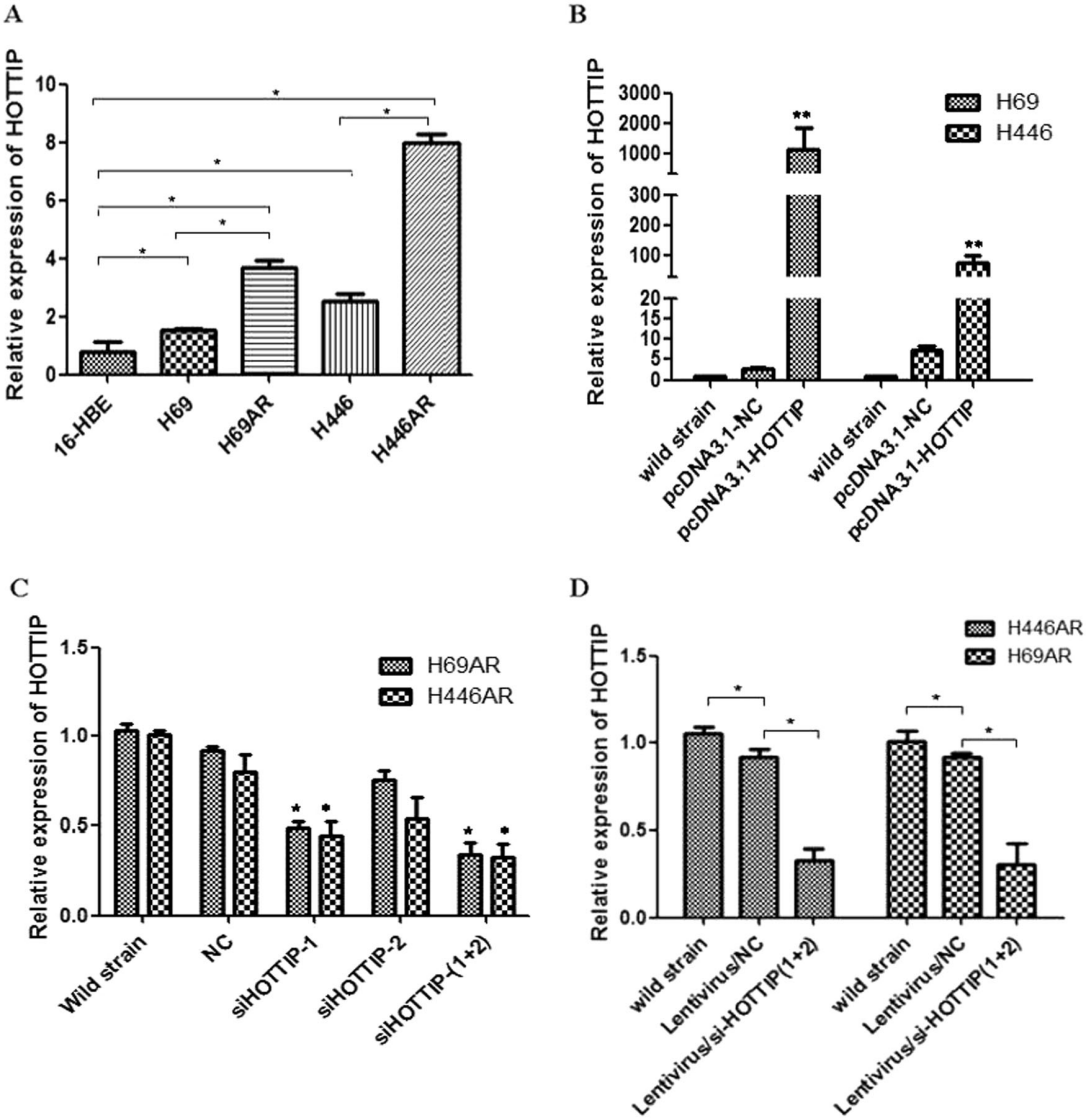
HOTTIP was upregulated in SCLC drug-resistant cell lines and establishment of HOTTIP overexpression and knockdown cell lines **a** Expression level of HOTTIP in four types of SCLC and 16-HBE cell lines show differential expression in SCLC cell lines, especially in drug-resistant cell lines, compared with expression in 16-HBE cell line. **b** Establishment of HOTTIP overexpression cell lines with a pcDNA3.1-HOTTIP expression vector being transfected into H69 and H446 cells, respectively. **c** Establishment of HOTTIP knockdown cell lines by siRNA method in H69AR and H446AR cells, respectively. **d** For stable HOTTIP RNAi effects, the RNAi sequences of si-HOTTIP-1 and si-HOTTIP-2 combination were packaged by lentivirus for the following studies, the transfection effects above were validated by electron fluorescence microscopy technique and RT-qPCR. *P < 0.05; **P < 0.001

We next performed RT-qPCR analysis to examine the expression levels of HOTTIP in several cell lines, including SCLC and the human bronchial epithelial cell lines (16-HBE) (Fig. 2a). Among the four SCLC cell lines investigated (H69, H69AR, H446, and H446AR), H69AR and H446AR expressed higher levels of HOTTIP than H69 and H446, respectively. Similarly, these four SCLC cell lines showed 1.962-, 4.6456-, 3.2278-, and 10.135-fold upregulation of HOTTIP over 16-HBE cell (Fig. 2a). To manipulate HOTTIP levels in SCLC cells, a pcDNA3.1-HOTTIP expression vector was presented abroad from Pro.WC^2^ and HOTTIP RNA interference (RNAi) sequences (GenePharma, Suzhou, China) were transfected into H69, H446, and H69AR, H446AR cells, respectively. RT-qPCR analysis of HOTTIP levels was performed at 24 h after transfection and revealed that HOTTIP expression was increased 437.62- and 10.77-fold in H69 and H446 cells, respectively, compared with negative control (NC) cells (Fig. 2b). However, in H69AR and H446AR cells, HOTTIP expression was effectively 44 and 11% knocked down by si-HOTTIP-1, 18 and 34% by si-HOTTIP-2, 66 and 67% by si-HOTTIP-1 and si-HOTTIP-2 combination RNAi effects, the latter combined small interfering RNAs (siRNAs) were subsequently used in the following loss-of-function studies of HOTTIP (Fig. 2c). For stable HOTTIP RNAi effects, the RNAi sequences of si-HOTTIP-1 and si-HOTTIP-2 combination were packaged by lentivirus for the following studies, the effects above were validated by electron fluorescence microscopy technique, RT-qPCR, and confocal microscopy both at mRNA and protein level (Fig. 2d).

### HOTTIP regulates apoptosis and chemoresistance of SCLC cells in vitro and in vivo

**Fig. 3:**
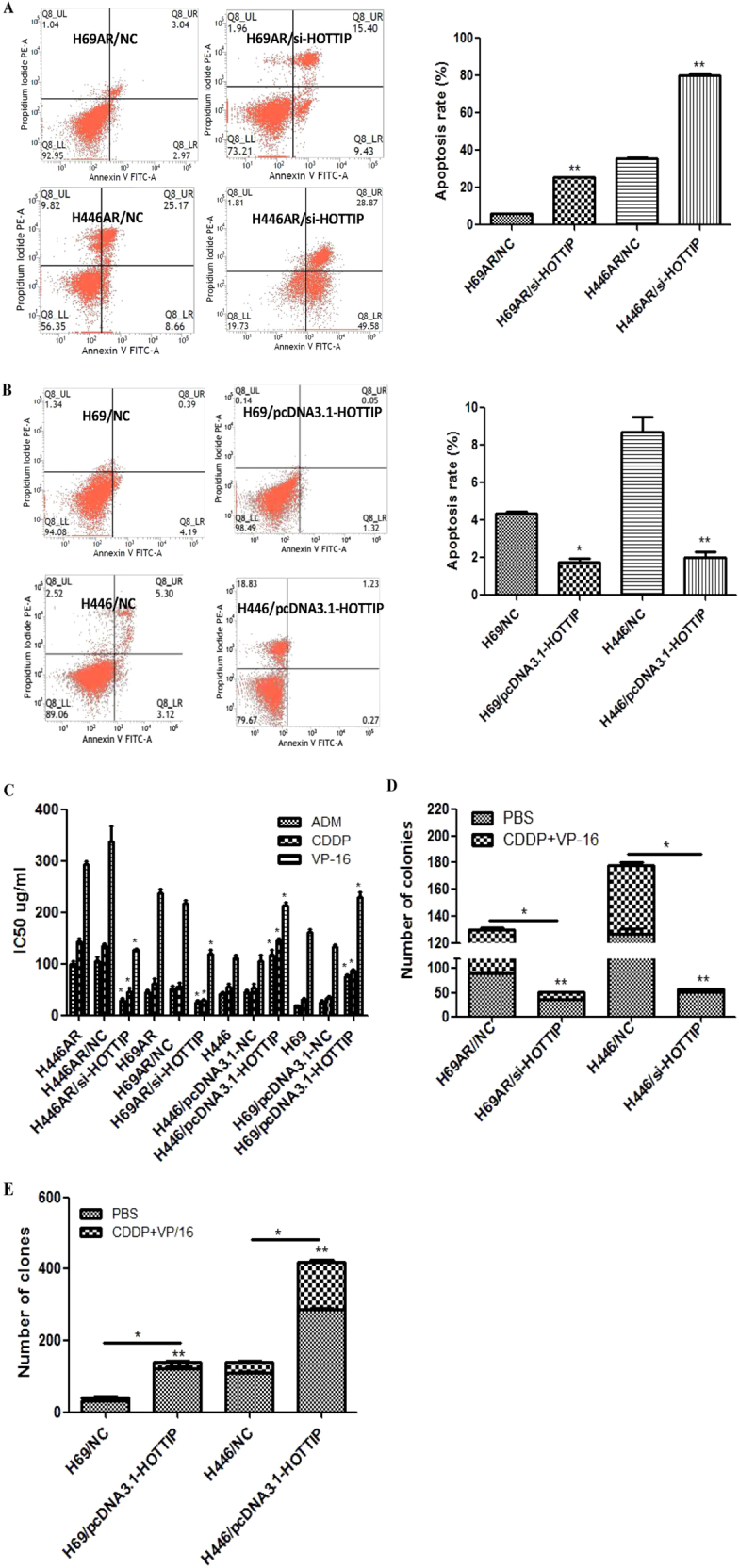
HOTTIP may promote SCLC cell apoptosis and chemoresistance in vitro **a**, **b** Flow cytometric analysis was used for cell apoptosis detection after HOTTIP knockdown and overexpression respectively. **c** CCK-8 assay was used to detect the effect of HOTTIP on SCLC cell chemoresistance after HOTTIP knockdown and overexpression, respectively. **d** Plate colony formation assay was used to detect the effect of HOTTIP knockdown on cell proliferation in vitro. **e** Plate and soft agar colony formation assay were used to detect the effect of HOTTIP overexpression on cell proliferation in vitro. *P < 0.05; **P < 0.01

To determine the effect of HOTTIP on chemoresistance in vitro, cell apoptosis assay was carried out in HOTTIP down- and upregulated cells. The result showed that the fraction of early apoptotic cells in HOTTIP knockdown cells was significantly higher than NC cells (Fig. 3a). Although the fraction of early apoptotic cells in HOTTIP overexpressed cells was significantly lower than NC cells (Fig. 3b). Next, we further tested whether HOTTIP affects the resistance of SCLC cells to adriamycin (ADM), CDDP, and VP-16. Results from cell counting kit-8 (CCK-8) assay showed that H69AR/si-HOTTIP and H446AR/si-HOTTIP cells exhibited much slower growth and a lower IC_50_ for drugs than NC cells. Although overexpression of HOTTIP resulted in much increased growth and a high IC_50_ for drugs (Fig. 3c, Table 2). Moreover, plate colony formation experiment showed that colony formation was significantly inhibited after either treatment with ADM, CDDP, and VP-16 or following HOTTIP knockdown (Fig. 3d). Although overexpression of HOTTIP led to a significant increase of colony number than either drugs treatment group or the control (Fig. 3e).

**Table 2:** Effect of HOTTIP on SCLC cell resistance index

	IC_50_ (μg/ml ±S)		
Cell groups	ADM	**CDDP**	VP-16
H69AR/si-HOTTIP	25.33 ± 5.51	29.0 ± 3.606	119.3 ± 15.01
H69AR/si-NC	52.0 ± 9.17	56.0 ± 11.53	216.67 ± 10.69
H69AR	45.67 ± 7.77	61.33 ± 16.50	237.3 ± 12.50
H446AR/si-HOTTIP	28.67 ± 6.51	46.33 ± 10.69	128.3 ± 2.89
H446AR/si-NC	104.67 ± 15.5	135.33 ± 5.69	337.67 ± 52.3
H446AR	98.33 ± 11.5	144.3 ± 10.07	292.33 ± 11.24
H69/pcDNA3.1+HOTTIP	75.33 ± 8.50	87.3 ± 3.51	229.00 ± 16.52
H69/pcDNA3.1+NC	25.67 ± 5.03	33.0 ± 7.21	134.33 ± 6.02
H69	19.0 ± 2.0	32.0 ± 3.0	161.33 ± 12.06
H446/pcDNA3.1+HOTTIP	117.3 ± 18.2	145.67 ± 7.09	214.33 ± 8.15
H446/pcDNA3.1+NC	45.0 ± 6.0	53.33 ± 12.42	105.0 ± 22.72
H446	42.3 ± 4.51	55.67 ± 11.06	111.67 ± 10.41

*ADM* adriamycin, *CDDP* cisplatin, *VP-16* etoposide

**Fig. 4:**
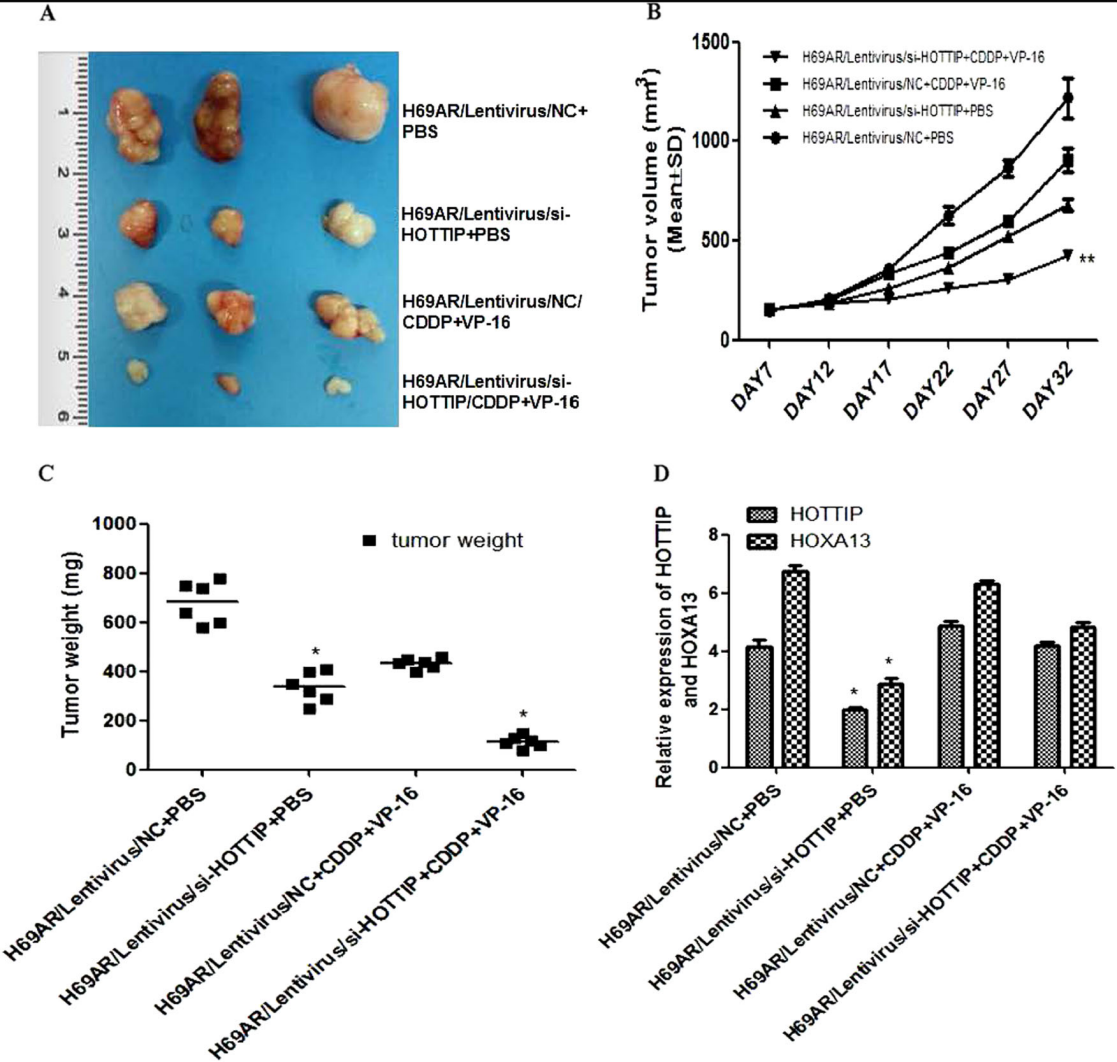
HOTTIP may promote SCLC cell chemoresistance and tumorigenesis in vivo **a** Excised tumors’ image from tumor bearing nude mice in each group. **b** Volume change curve of each group measured on the indicated days. **c** Tumor weights of each group were determined. **d** Relative expression of HOTTIP and HOXA13 mRNA in nude mice tumor tissues by RT-qPCR. *P < 0.05; **P < 0.01

The ability of HOTTIP to confer chemoresistance was further examined using an in vivo tumor model. As shown in Fig. 4a, compared with the control group, HOTTIP knockdown resulted in a smaller size of the subcutaneous tumor in mice, and intraperitoneal injection of CDDP + VP-16 into the mice with HOTTIP knockdown further inhibited the growth of the tumor. After treating for 17 days, the mean tumor volume for H69AR/Lentivirus/si-HOTTIP + CDDP + VP-16 group was more and markedly smaller than other groups (Fig. 4b). As expected, the weight statistic of excised tumor showed a similar trend to tumor volume (Fig. 4c). However, after intermittent administration of chemotherapy drugs for several weeks, HOTTIP and HOXA13 expression in Lentivirus/si-HOTTIP + CDDP + VP-16 group is significantly higher than Lentivirus/si-HOTTIP + phosphate-buffered saline (PBS) group, whereas the difference of their expression in Lentivirus/NC + CDDP + VP-16 and Lentivirus/si-HOTTIP + CDDP + VP-16 group was significantly reduced (Fig. 4d).

### HOXA13 partly mediates the effect of HOTTIP on SCLC chemoresistance and correlated with SCLC prognosis

**Fig. 5:**
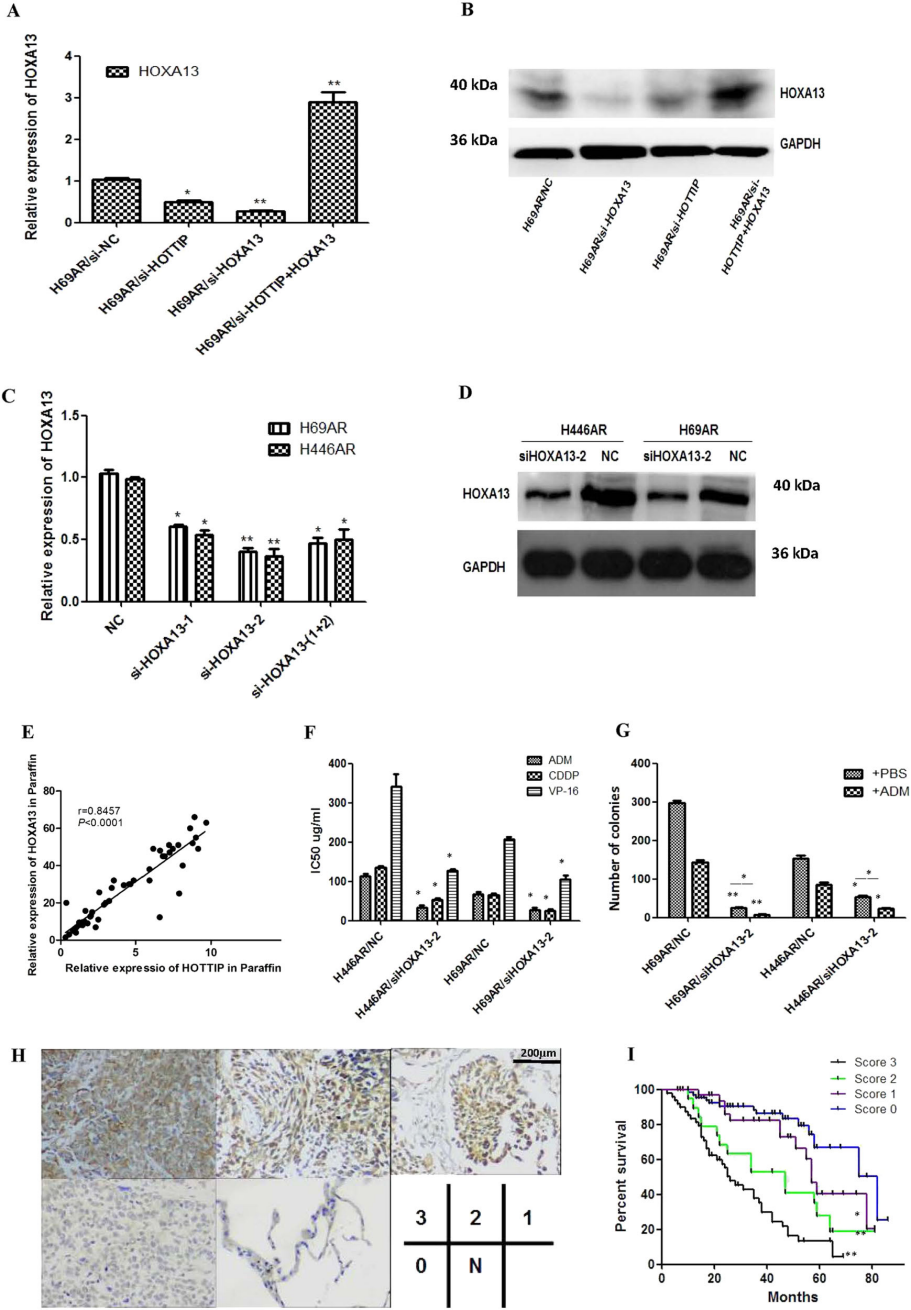
HOXA13 expression was regulated positively by HOTTIP and correlated with SCLC prognosis **a**, **b** HOTTIP knockdown may reduce expression of HOXA13 mRNA and protein in H69AR and H446AR cells, whereas HOXA13 overexpression may rescue the HOXA13 suppression caused by HOTTIP knockdown. **c**, **d** Knockdown of HOXA13 was successful both at mRNA and protein levels in H69AR cell. **e** Correlation scatterplot (Spearman test) between HOTTIP and HOXA13 expression levels was detected in clinical samples. **f**, **g** The effect of HOXA13 on cell chemoresistance and proliferation were observed by CCK-8 assay and plate clone formation. **h** The relationship between HOXA13 expression and clinical pathological characteristics was detected by immunohistochemistry. **i** Survival differences between groups with different scores were statistically assessed by the Kaplan–Meier method and log-rank test. *P < 0.05; **P < 0.01

For the gene expression array analysis in Supplementary Figure 1C, both HOTTIP and HOXA13 gene were upregulated >10-fold changes in H69AR compared with H69 cell line. Based on the physical proximity of HOXA gene cluster and HOTTIP^2^, we further detected the expression of HOXA genes in two strains of cells, the results showed that HOXA13 was significantly higher than the other HOXA genes (Supplementary Figure 1E). As emerging evidence suggests that certain members of the HOXA cluster are involved in cancer progression, we hypothesized that HOTTIP might regulate the biological behavior of SCLC via regulation of the HOXA cluster. To confirm this hypothesis, we first evaluated the effect of HOTTIP knockdown on the expression of 5’ HOXA genes (HOXA13, HOXA11, HOXA10, and HOXA9) by RT-qPCR in H69AR and H69 cells, and found that knockdown of HOTTIP inhibited the expression of these genes to varying degrees, with the strongest inhibition observed for HOXA13 (Supplementary Figures 2A-2B). As HOXA13 has been reported to be more closely related with HOTTIP in hepatocellular carcinoma^13^, we then confirmed their relationships in SCLC chemoresistance. Rescue experiment by HOXA13 overexpression demonstrated that HOTTIP may regulate HOXA13 expression (Figs. 5a, b). Further evaluation showed a positive correlation between HOTTIP and HOXA13 expression in clinical samples, which confirms the association between HOTTIP and HOXA13 expression we observed above (Fig. 5e).

To further investigate the role of HOXA13 in SCLC pathogenesis and chemoresistance, we used siRNA technique and found siHOXA13-2 more effective by RT-qPCR and western blot (Figs. 5c, d). Interestingly, downregulation of HOXA13 also inhibited cell proliferation and chemoresistance (Figs. 5f, g), which is consistent with biology function of HOTTIP. To determine the clinical relevance of the HOTTIP/HOXA13 axis in SCLC, immunohistochemistry (IHC) method was used to detect HOXA13 expression in SCLC tissues and indicated that, only 3 of 60 cases were designated a score of 0 (negative staining), and higher staining intensity of HOXA13 may be correlated with extensive disease stage, poorer chemotherapy response, shorter median survival time, and poorer prognosis of SCLC patients (Supplementary Table 2, Figs. 5h, i). These results above imply that HOTTIP acts at least partly by controlling HOXA13 in SCLC poor prognostic and chemoresistance progression.

### HOTTIP may act as sponge of miR-216a and enhanced the expression of its another target gene, anti-apoptotic gene BCL-2

**Fig. 6:**
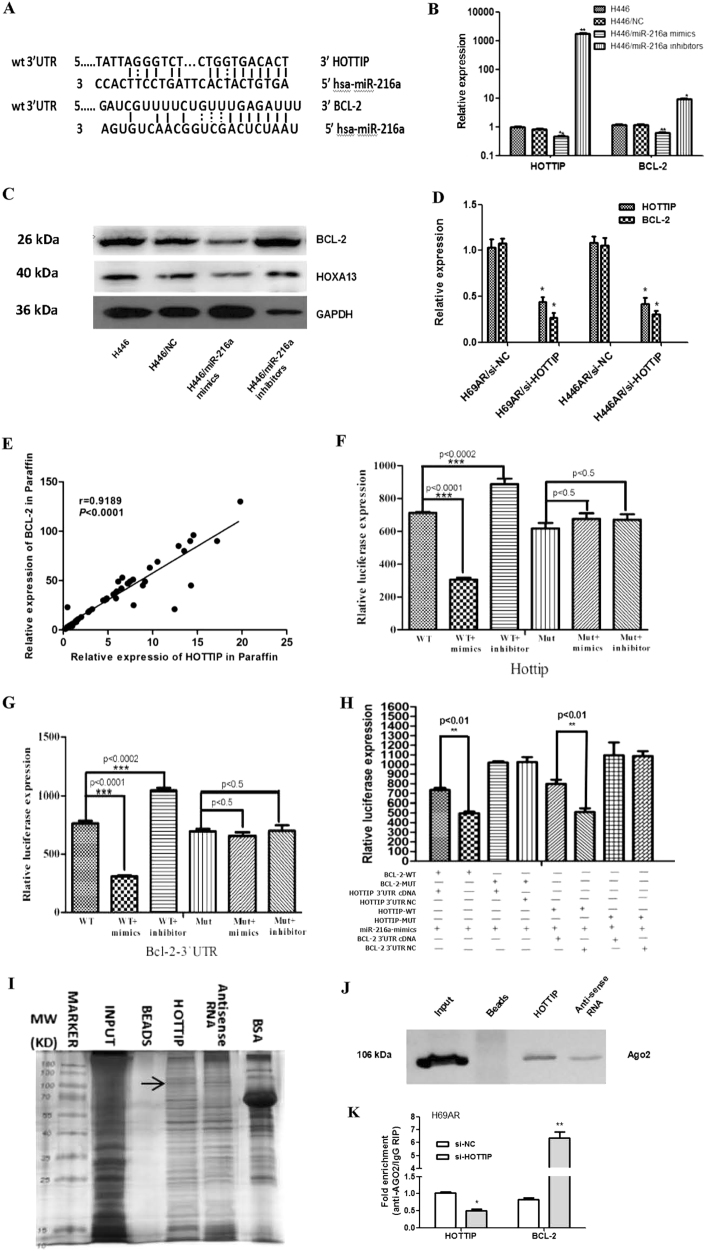
HOTTIP may promote SCLC chemoresistance and pathogenesis through ceRNA mechanism **a** Putative binding site of miR-216a in HOTTIP and BCL-2 3’-UTR and the site of target mutagenesis are indicated. **b** miR-216a may negatively regulated expression of its target genes HOTTIP and BCL-2. **c** miR-216a may negatively regulated expression of HOXA13 and BCL-2 proteins. **d** HOTTIP may positively regulated expression of BCL-2 in H69AR cell. **e** The relevance analysis of HOTTIP and BCL-2 expression in SCLC FFPE tissues. **f**–**h** Luciferase activity of the indicated group in H69AR cell. **i, j** RNA pull-down, mass spectrometry and western blot showed a possible interaction exists between HOTTIP and Ago2. **k** RIP assay of the enrichment of Ago2 on HOTTIP and BCL-2 transcripts relative to IgG in H69AR cell transfected with si-HOTTIP. *P < 0.05; **P < 0.01; ***P < 0.001

There were 81 miRNAs related closely to SCLC biology by miRNA microarray, among them 37 miRNAs expressed higher in H69 cell compared with H69AR cell, whereas 44 miRNAs expressed lower in H69 cell compared with H69AR cell (Supplementary Figure 2C, detailed data not shown). miR-216a were screened out from 28 miRNAs after miRNA microarray for being related closely to SCLC chemoresistance (Supplementary Figures 2D-2E, Supplementary Table 3), and it was predicted by the bioinformatics website RNA22-seq (https://cm.jefferson.edu/) that it has targeted binding relationship with HOTTIP (Fig. 6a). Among the potential targeted genes of miR-216a, we surprisingly found the apoptosis-related gene, BCL-2 (Fig. 6a). Then, we verified their predicted target regulation relationship by RT-qPCR and western blot in H446 cell and found that, miR-216a acts as an inhibitor of BCL-2, HOTTIP, and HOXA13 at either mRNA or protein level (Figs. 6b, c). Moreover, by knockdown HOTTIP expression in H69AR and H446AR cells, BCL-2 was significantly decreased correspondingly (Fig. 6d). We detected co-expression relationship of BCL-2 and HOTTIP in FFPE tissues as well, and found a positive correlation was shown between their expression (*r* = 0.9189, *P* < 0.0001; Fig. 6e).

To further investigate the regulating relationship of miR-216a, HOTTIP, and BCL-2, we performed the HOTTIP and BCL-2 plasmids co-transfection luciferase reporter assay. The results showed that luciferase activity was reduced by 58.3% for HOTTIP (Fig. 6f) and 62.5% for BCL-2 (Fig. 6g) as compared with their empty vector control when miR-216a were expressed, respectively. These data demonstrate that miR-216a can directly bind to HOTTIP and BCL-2, respectively, through their own miRNA recognition sites. Although co-transfection luciferase reporter assay reveled that, in the presence of HOTTIP, the inhibited reporter plasmid luciferase activity for BCL-2 was reversed (Fig. 6h), which confirmed that HOTTIP can compete with BCL-2 to binding to miR-216a. This indicates that HOTTIP acts as an endogenous “sponge” by binding miR-216a, thus abolishing the miRNA-induced repressing activity on the BCL-2 3’-UTR. Together these data indicate that by binding miR-216a, HOTTIP acts as a competitive endogenous RNA (ceRNA) for the target BCL-2 mRNA, thereby modulating the derepression of BCL-2 and imposing an additional level of post-transcriptional regulation.

As we all know, miRNAs are present in the form of miRNA ribonucleoprotein complexes within the cytoplasm, there the core component of RNA-induced silencing complex (RISC), Ago2 was involved^19^. To detect whether HOTTIP is present in miRNPs microRNA-containing ribonucleoprotein complexes (miRNPs) RNA pull-down, mass spectrometry and western blot assay were carried out and showed a possible direct interaction between HOTTIP and Ago2 (Figs. 6i, j, Supplementary Table 4). Moreover, RIP assay on Ago2 was performed, which is the vital component of the RISC. As shown in Fig. 6k, knockdown of HOTTIP led to the decreased enrichment of Ago2 on HOTTIP, but substantially increased enrichment on BCL-2 transcripts. These results indicate that HOTTIP could compete with BCL-2 transcripts for the Ago2-based RISC.

### miR-216a suppresses SCLC cells chemoresistance and correlates with prognosis

**Fig. 7:**
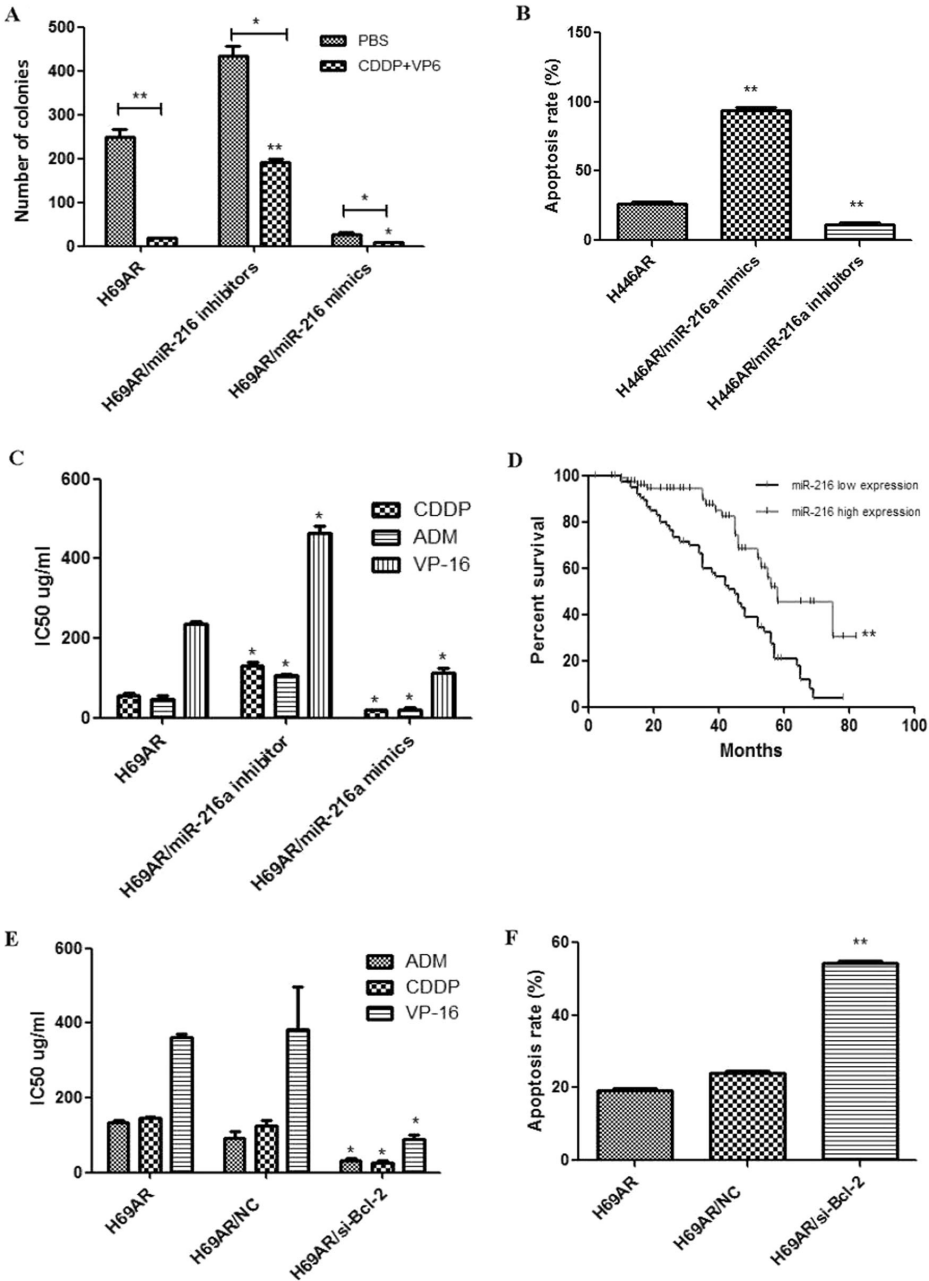
miR-216a suppresses SCFLC chemoresistance, enhances cell apoptosis by targeting to BCL-2, and correlates with prognosis of SCLC patients **a** Plate clone formation assay showed miR-216a may inhibit SCLC cell proliferation and chemoresistance. **b** miR-216a may enhance SCLC cells apoptosis. **c** Effects of miR-216a knockdown and overexpression on SCLC cells chemoresistance. **d** miR-216a may correlate with prognosis of SCLC patients. **e** Effects of BCL-2 knockdown on SCLC cells chemoresistance. **f** BCL-2 knockdown may enhance SCLC cells apoptosis. *P < 0.05; **P < 0.01

To verify whether miR-216a might also suppress SCLC cells chemoresistance, we forced and knockdown miR-216a expression in H69AR cells with miRNA-encoding plasmids transfection. CCK-8 assay, plate colony formation experiment, and flow cytometry were carried out to detect chemoresistance, viability, and apoptosis rate of SCLC cells affected by miR-216a. The results revealed that cells transfected with miR-216a mimics showed more chemosensitive, lower colony forming rate, and higher apoptosis rate compared with wild-type (WT) cells, whereas cells transfected with miR-216a inhibitors showed an opposite result (Figs. 7a–c). To further investigate whether miR-216a may influence prognosis of the patients, we detected the expression of miR-216a in FFPE tissues and found that, the patients with higher miR-216 lever got a longer survival stage, which suggest miR-216a might act as an SCLC prognostic factor (Fig. 7d). According to the targeting regulation relationship between miR-216a and BCL-2, we further investigated whether BCL-2 was involved in SCLC chemoresistance and cell apoptosis, and the results showed that BCL-2 knockdown exactly enhance sensitivity to chemotherapy drugs (Fig. 7e) and H69AR cell apoptosis (Fig. 7f).

## Discussion

The prognosis of SCLC remains extremely poor mainly for recurrence and chemoresistance, but the mechanism of chemoresistance is still unclear^20^. It has been reported that lncRNAs were involved in anticancer drug resistance^19,21–25^. For example, lncRNA UCA1 was found to decrease chemosensitivity in bladder cancer by activating Wnt signaling^25^, and lncRNA MEG3 increases CDDP resistance in lung adenocarcinoma^22^. lncRNA long non-coding RNA regulator of reprogramming (ROR) regulates chemoresistance of human hepatocellular cancer cell and so on^24^. To investigate whether lncRNA is involved in SCLC chemoresistance, we performed lncRNA microarray to compare the comprehensive lncRNA expression profiles in SCLC chemoresistance cells. As HOTTIP is one of the most upregulated genes (>10-fold changes) in H69AR as compared with H69 cell line and has not been reported in SCLC, so HOTTIP was chosen for the further study.

HOTTIP, which lies at the 5′ tip of the HOXA locus and coordinates the activation of multiple 5′HOXA genes in vivo, has been identified as one of 231 lncRNAs associated with the human HOX loci^26^. This lncRNA directly binds the adaptor protein WDR5 and targets WDR5/MLL complexes across HOXA, thus driving histone H3 lysine 4 trimethylation and gene transcription^2^. Furthermore, HOTTIP has been identified as a negative prognostic factor in hepatocellular carcinoma patients^13^. However, whether HOTTIP was involved in SCLC chemoresistance remains unknown.

To investigate the role of HOTTIP in SCLC chemoresistance, PCR-based gene expression profiling array was performed in paired human SCLC cell line H69 and its drug resistance cell line H69AR, the results showed a differential expression of 16 HOX family members, among which HOXA13 gene and lncRNA HOTTIP that is on the physical proximity of HOXA gene were upregulated most remarkably in H69AR cell. It is exciting that they are the subjects of our interest. Thus, a significant differential expression of HOTTIP and HOXA13 gene were screened out among the 1443 genes, and was invalided later both in cell lines and clinical samples, which indicates a close correlation between HOTTIP and HOXA13 gene and SCLC cell chemoresistance, as well as clinical outcome. Furthermore, for loss and gain of HOTTIP function studies, targeted silencing and overexpression of HOTTIP could potentiate or weaken the anti-chemoresistance effects of ADM, CDDP, and VP-16 both in vitro and in vivo. Moreover, HOTTIP was revealed to regulate SCLC biology by mediating HOXA13 expression in series of experiments. However, HOTTIP downregulation may impair the expression of endogenous HOXA13 protein, whereas transfection of HOXA13 plasmids increased expression of exogenous HOXA13 protein, so rescue experiment by HOXA13 overexpression at least cause the increase of HOXA13 expression, not only because the HOXA13 plasmid encode for its coding sequence. On the other hand, an interesting phenomenon seems to increase the value of our research, that is, after intermittent administration of chemotherapy drugs for several weeks, HOTTIP and HOXA13 expression in Lentivirus/si-HOTTIP + CDDP + VP-16 group is significantly higher than Lentivirus/si-HOTTIP + PBS group, whereas the difference of their expression in Lentivirus/NC + CDDP + VP-16 and Lentivirus/si-HOTTIP + CDDP + VP-16 group was significantly reduced. For this phenomenon, we hypothesized that, tumor-bearing mice may be tolerated in response to the intermittent administration of chemotherapy, and the expression of HOTTIP and HOXA13 in the tumors showed a corresponding increase.

Enlightened from the reported ceRNAs regulatory mechanism, and lots of emerging evidence have shown that lncRNAs may be involved in the regulatory network^12,27,28^. To investigate whether HOTTIP can play a role as ceRNA in SCLC chemoresistance, we searched for its potential interactions with miRNAs by bioinformatic analysis. As expected, has-miR-216a was predicatively discovered to form complementary base pairing with HOTTIP and another popular apoptosis-suppressing gene, BCL-2 unexpectedly. By luciferase reporter assay, we confirmed miR-216a could directly bind to HOTTIP and BCL-2 simultaneously, and co-transfection luciferase reporter assay reveled that HOTTIP could reverse the inhibited reporter plasmid luciferase activity for BCL-2, which confirmed that HOTTIP can compete with BCL-2 to bind with miR-216a. Furthermore, HOTTIP and miR-216a coimmunoprecipitation (RIP) with anti-Ago2 showed a positive physical interaction in SCLC cells, which provides further support for HOTTIP’s miRNA-sponging activity. Furthermore, RT-qPCR assay demonstrated miR-216a expression was lower in H69AR and H446AR cells, whereas HOTTIP expression in these two cell lines is higher, which verifies the truth of HOTTIP act as endogenous “sponge” for miR-216a. Moreover, ectopic low expression of miR-216a could promote SCLC proliferation and chemoresistance in H69AR and H446AR cells, which was consistent with effects of HOTTIP overexpression in H69 and H446 cells. Taken together, our data present, for the first time, that HOTTIP functions as a ceRNA via competing for miR-216a to regulate Bcl-2 expression in SCLC chemoresistance improvement.

Finally, the findings presented in this study have allowed us to conclude that HOTTIP overexpression represents a novel biomarker of poor prognosis in SCLC, and may confer multiple properties required for tumor progression and chemoresistant phenotype. Although the pro-oncogenic role of HOTTIP has been reported in some cancers including pancreatic cancer, non-SCLC, colorectal cancer, and so on^6–11^. However, its role and the mechanism in SCLC has not been reported yet. More importantly, our study indicates that the ceRNA activity of HOTTIP imparts a miRNA/lncRNA trans-regulatory function to protein-coding mRNAs and the ceRNA network may play an important role in SCLC chemoresistance pathogenesis. In all, our experimental data suggest that targeting the HOTTIP–BCL-2 interaction may represent a novel therapeutic application, thus contributing a better knowledge to choose the combination therapy based on chemotherapy and combining with biological therapy for BCL-2 positive SCLC patients.

## Materials and methods

### Computational analysis

Two human lncRNA microarray data sets (GSE55191, http://www.ncbi.nlm.nih.gov/geo/query/acc.cgi?acc=GSE55191 and GSE58043, http://www.ncbi.nlm.nih.gov/geo/query/acc.cgi?acc=GSE58043) were obtained from public database NCBI^29,30^. Aberrantly expressed lncRNAs were identified using Venn analysis and co-expression network analysis^31^.

### Human tissue specimens and cell culture

A total of 115 FFPE tissues were obtained from patients who had underwent bronchofiberscopy or biopsy for SCLC diagnosis during January 2008 to January 2015 and receiving care and follow-up at Southern Medical University affiliated Shunde people’s hospital. The non-cancerous lung tissues in our study are all from the lung benign diseases including bronchiectasis and pulmonary bulla by thoracoscopic lobectomy. Informed consent was obtained from all patients and the study was approved by the Hospital’s Protection of Human Subjects Committee. Clinical data included the patient gender, age, smoking history, limited-, or extensive-stage disease and follow-up (Table 1).

H69, H69AR, H446, H446AR SCLC cell lines and 16-HBE alveolar epithelial cell line were obtained from the American Type Culture Collection (ATCC, USA), cultured in RPMI-1640 medium containing 10 and 20% fetal bovine serum in a humidified incubator at 37 °C with 5% CO_2_, respectively. These SCLC cell lines were purchased from ATCC, and H69AR cell was established by Mirski et al.^32^ through induced resistance of H69 cells to ADM intermittently, they are both mainly used in studies of SCLC drug resistance mechanism. The ADM-resistant NCI-H446 cell line (NCI-H446AR) was also obtained by culturing H446 cell in gradually increasing doses of ADM up to 0.8 µM after a total of 14 months in our laboratory^33^. The drug-resistant cells including H446AR or H69AR cell line were maintained in drug-free medium for at least 2 weeks before any experiment.

### Microarray analysis

Transcriptomic analysis was performed by human lncRNA microarrays (Arraystar Company, USA), which target differential expression of 1443 lncRNAs on H69AR cell and H69 cell. Differentially expressed genes with an raw expression level of >400 and ordered by *P*-value. Genes with the highest top 10 *P*-values were selected for validation. The detailed experimental procedures were performed as previously described^34^.

### Establishment of H446AR cell line

As mentioned above, as NCI-H446 cells was cultured in gradually increasing doses of ADM for 8 months, H446AR cells, which could continuously grow in 0.4 µM ADM were obtained^22^. The remaining process is established by referring establishment of H69AR^32^.

### Overexpression and RNA interfere

The overexpression plasmid pcDNA3.1-HOTTIP was given as a present from Professor Kevin Wang^2^. The pcDNA3.1-NC plasmid, siRNAs/shRNAs, and miRNA mimics/inhibitors/antagomirs were purchased from GenePharma (Shanghai, China). The effective interference sequences were all selected by RT-PCR for the best gene silencing effect and then used for subsequent experiments. For stable transfection, positive transfectants were selected with 400 µg/ml G418 (Calbiochem), whereas HOTTIP siRNAs were packaged by lentivirus. The related siRNAs, shRNAs, or miRNA mimics/inhibitors/antagomirs sequences were listed in Supplemental material.

### RNA isolation and RT-qPCR

RT-qPCR was used to detect expression levels of HOTTIP and other genes in SCLC cancer tissues and cells according to the manufacturer’s instructions. Total RNA, including miRNAs, was extracted from cells, nude tumor tissues, and FFPE tissues using Trizol reagent (Invitrogen) and miRNeasy FFPE Kit (Qiagen) according to the manufacturer’s protocols. According to Prime Script RT reagent Kit (TIANGEN, Beijing, China), reverse transcription reactions were processed at 42 °C for 15 min, followed by 3 min at 95 °C for complementary DNA synthesis. Then quantitative reverse transcription-PCR was performed in an ABI illumina instrument. The miRNA sequence-specific reverse transcription qRT-PCR for miR-216a and endogenous control U6 were performed according to Hairpin-it^TM^ miRNAs qRT-PCR quantization kit and U6 snRNA real-time PCR normalization kit (GenePharma, Shanghai, China). Glyceraldehyde 3-phosphate dehydrogenase or U6 was used as endogenous controls. Fold changes were calculated using relative quantification (2^−ΔΔCt^) method.

### Western blot analysis

Western blot analysis were performed according to standard protocols as described previously^1^. All antibodies information are listed in Supplementary Information.

### IHC staining analysis

IHC staining analysis were performed according to standard protocols as described previously^1^. All antibodies information are listed in Supplementary Information.

### Immunofluorescence analysis

Cells were seeded and fixed on 12 × 12 mm glass slides. For intracellular staining (HOXA13), the cells were fixed with 4% paraformaldehyde and permeabilized by incubation with 0.5% Triton X-100 for 2 min. The cells were incubated with 5% non-fat milk for 15 min at room temperature. After washing with PBS for 3 × 5 min, the cells were incubated with specific primary antibody of HOXA13 at 4 °C overnight. Antibody dilutions of 1:100 were used for HOXA13. The cells were then washed and incubated with Alexa Fluor 633-conjugated goat anti-rabbit IgG for 1 h. The nuclei were then stained with 4,6-diami-dino-2-phenylindole. Sections were then visualized by a FV1000 confocal microscope (Olympus Corp, Tokyo, Japan).

### In vitro chemosensitivity assay

For CCK-8 assay, cells were plated in 96-well plates at 5 × 10^3^ cells per well. After adherence of stable transfected cells, cells were treated with three kinds of chemotherapy drugs (cisplatin (CDDP, Shandong, China), VP-16 (Jiangshu, China), ADM (Jiangshu, China)), respectively, for 24 h. The absorbance at 450 nm was measured after incubation with 10 μl of CCK-8 reagent (Dojindo, Kumamato, Japan) for 4 h. The cells incubated without drugs were set at 100% survival and were used to calculate the concentration of each chemotherapeutic drug IC_50_. The assay was conducted in five replicate wells for each sample and three parallel experiments were performed.

For plate colony formation, the stable transfected cells were seeded (500 cells per well) in six-well plates overnight and chemotherapy drugs (Cisplatin (CDDP; China), VP-16 (China), ADM (China)) (4 μM) or PBS (100 μl) was added to the cultured cells on the second day. After 14 days, the culture medium was removed, and cells were briefly rinsed with PBS. The cells were then fixed with 4% paraformaldehyde and stained with 0.1% crystal violet, and colonies were counted by visual inspection.

### In vivo tumor formation and chemosensitivity assays

Tumor formation experiment in BALB/c mice was carried out according to the institutional guidelines of Guangdong Province and being approved by the Use Committee for Animal Care. Twenty-four BALB/c nude mice (male, 5–6 weeks old, 18.0 ± 0.5 g) were obtained from the Guangdong Medical Animal Center. This experiment was carried out at the Animal Experimental Department of Sun Yat-sen University North District. They were randomly divided into the following groups (*n* = 6 mice per group): (a) H69AR/lentivirus-NC, PBS; (b) H69AR/lentivirus-si-HOTTIP, PBS; (c) H69AR/lentivirus-NC, CDDP + VP-16; (d) H69AR/lentivirus-si-HOTTIP, CDDP + VP-16. H69AR cells were harvested and re-suspended in serum-free medium at a concentration of 1 × 10^7^ cells per 0.2 ml. Each mouse was injected subcutaneously in the flanks with cells, above injection the mice also received chemotherapy drugs (CDDP (China) 5 mg/kg and VP-16 (China) 2.5 mg/kg) or PBS (100 μl) intraperitoneal (i.p.) injection after the tumor volume was over 100 mm^3^ twice a week for five times totally. The tumors were measured every 3–4 days, and tumor volume was calculated using the following formula: volume = (L × W^2^)/2, and L and W are the longest and shortest diameters, respectively. The mice were sacrificed when the average L of any group reached approximately 1 cm.

### Apoptosis analysis by flow cytometry

For apoptosis assay, cells were transfected with si-HOTTIP and pcDNA3.1-HOTTIP plasmid, respectively, then all cells groups were treated with chemo-drugs for 24 h before being collected. Annexin V/propidium iodide detection kit (Keygene, Nanjing, China) was used for cell apoptosis assay. As there were spontaneous green fluorescence with cells after transfection, so the gate in detecting by flow cytometry should be regulated in cell apoptosis with negative staining and blank control.

### Luciferase reporter assay

Using dual luciferase assay kit (Beyotime Biotechnology) for dual luciferase assay. psiCHECK2.0 plasmid encoding a luciferase reporter gene was purchased from Promega. Recombinant plasmid of psiCHECK2.0-H-HOTTIP-3’-UTR, psiCHECK2.0-H-Bcl2-3’-UTR (WT) or corresponding mutant type (Mut) was constructed in GenePharma (Suzhou, China). H69AR cells (1–2 × 10^5^ cells per well) were plated in a 12-well plate and co-transfected with 40 nM of either hsa-miR-216a-5p or miRNA NC of either recombinant plasmids or corresponding mutants, and 1 ng of psiCHECK2.0 (Promega) by using Lipofectamine^TM^ 2000. The psiCHECK2.0 vector was used as an internal control to correct the differences in both transfection and harvest efficiency. H69AR cells were collected 48 h after transfection and analyzed.

### RNA pull-down assay

Briefly, biotin-labeled RNAs were transcribed in vitro with the Biotin RNA Labeling Mix (Roche) and T7 RNA polymerase (Roche), treated with RNase-free DNase I (Roche), and purified with the RNeasy Mini Kit (Qiagen). The procedure was carried out according to the manufacturer’s instructions and standard protocols as described previously^35^.

### Mass spectrometry

HOTTIP and antisense strand protein bands acquired by RNA pull-down assay were excised and examined by mass spectrometry to detect the related protein combined directly with HOTTIP. The procedure was carried out according to standard protocols described previously^35^.

### RNA immunoprecipitation (RIP) assay

RIP was conducted using Magna RIP RNA-Binding Protein Immunoprecipitation Kit (Millipore) following the manufacturer’s protocol.

### Statistical analysis

All experiments were run in triplicate. Data were represented as mean ± SD. All statistical analyses were carried out using GraphPad Prism 5 Software. Statistical significance was analyzed by Student’s *t*-test or one-way analysis of variance. The association between HOTTIP expression and clinical features were analyzed by Fisher’s exact test. Differences in patient survival were assessed using the Kaplan–Meier method and analyzed using the log-rank test in an univariate analysis. *P < *0.05 was considered significant.

## Electronic supplementary material

Supplementary Table 1

Supplementary Table 2

Supplementary Table 3

Supplementary Table 4

Supplementary information

Supplementary Figure 1

Supplementary Figure 2
