# Supplementary figures and images for: Long non-coding RNA HOTTIP promotes BCL-2 expression and induces chemoresistance in small cell lung cancer by sponging miR-216a
Source: Cell Death Dis. 2018 Jan 24;9(2):85. doi: 10.1038/s41419-017-0113-5 (PMC5833383; doi:10.1038/s41419-017-0113-5)

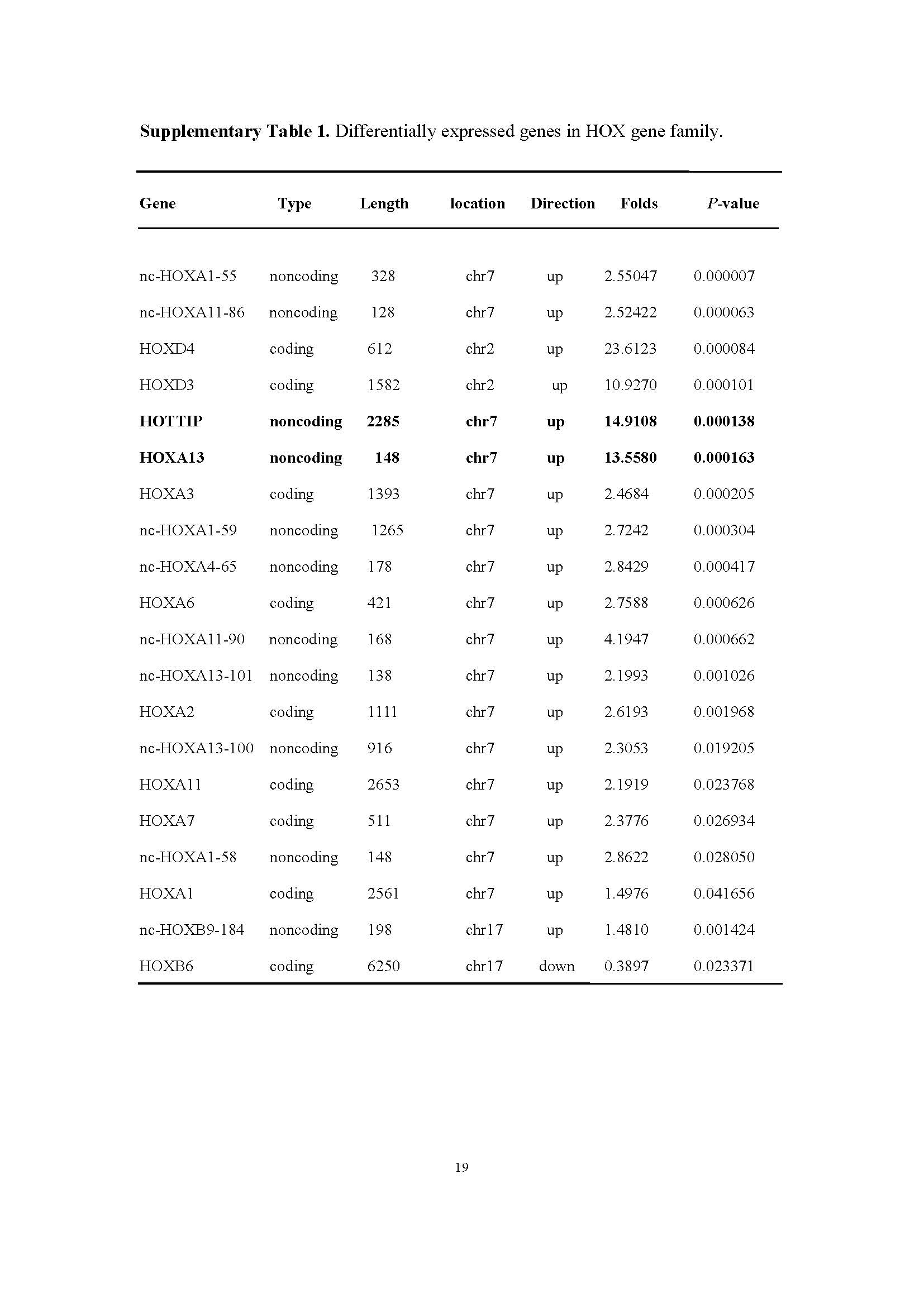

Supplement: Supplementary file 1 — Supplementary Table 1 [file 41419_2017_113_MOESM1_ESM.jpg]

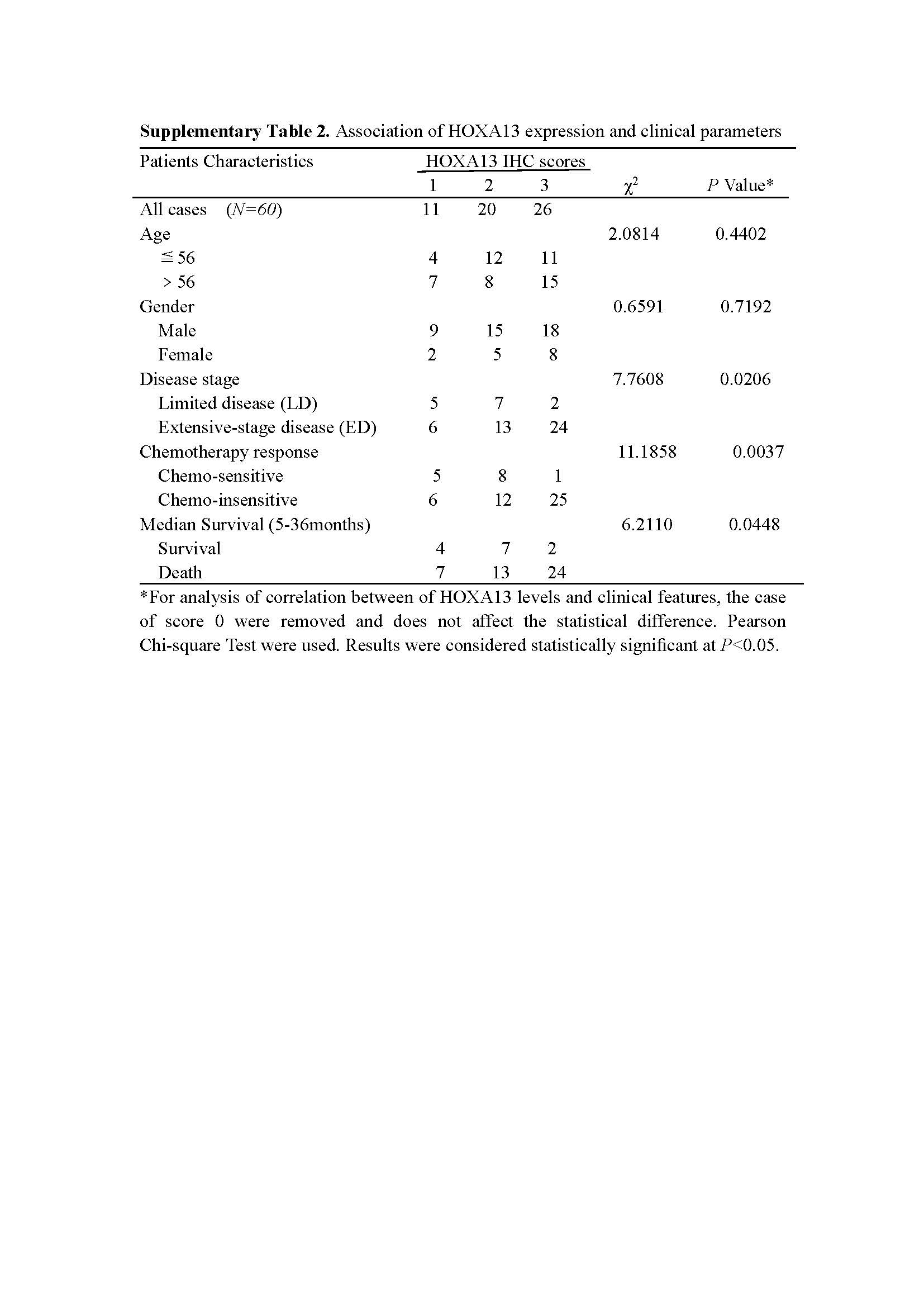

Supplement: Supplementary file 2 — Supplementary Table 2 [file 41419_2017_113_MOESM2_ESM.jpg]

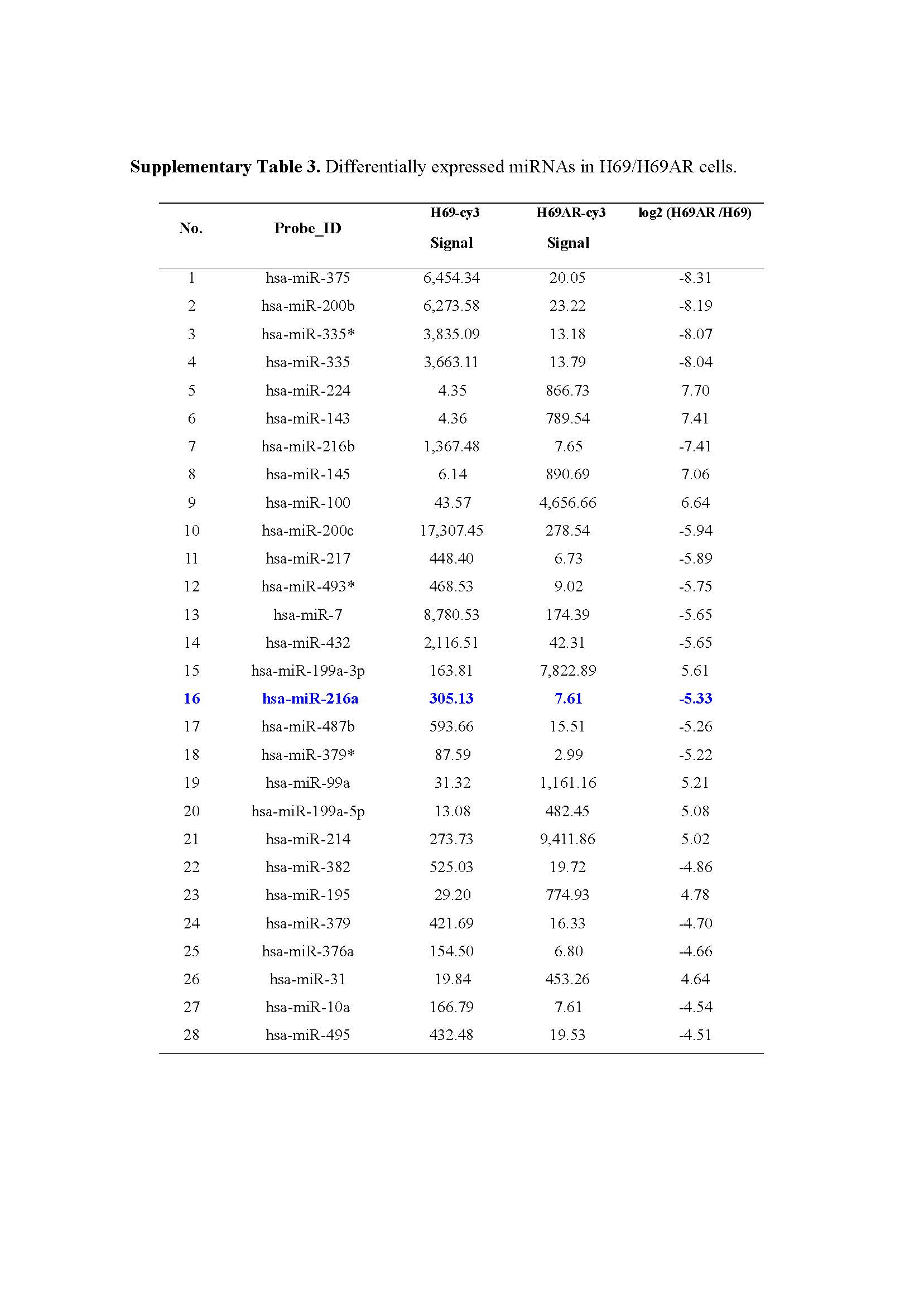

Supplement: Supplementary file 3 — Supplementary Table 3 [file 41419_2017_113_MOESM3_ESM.jpg]

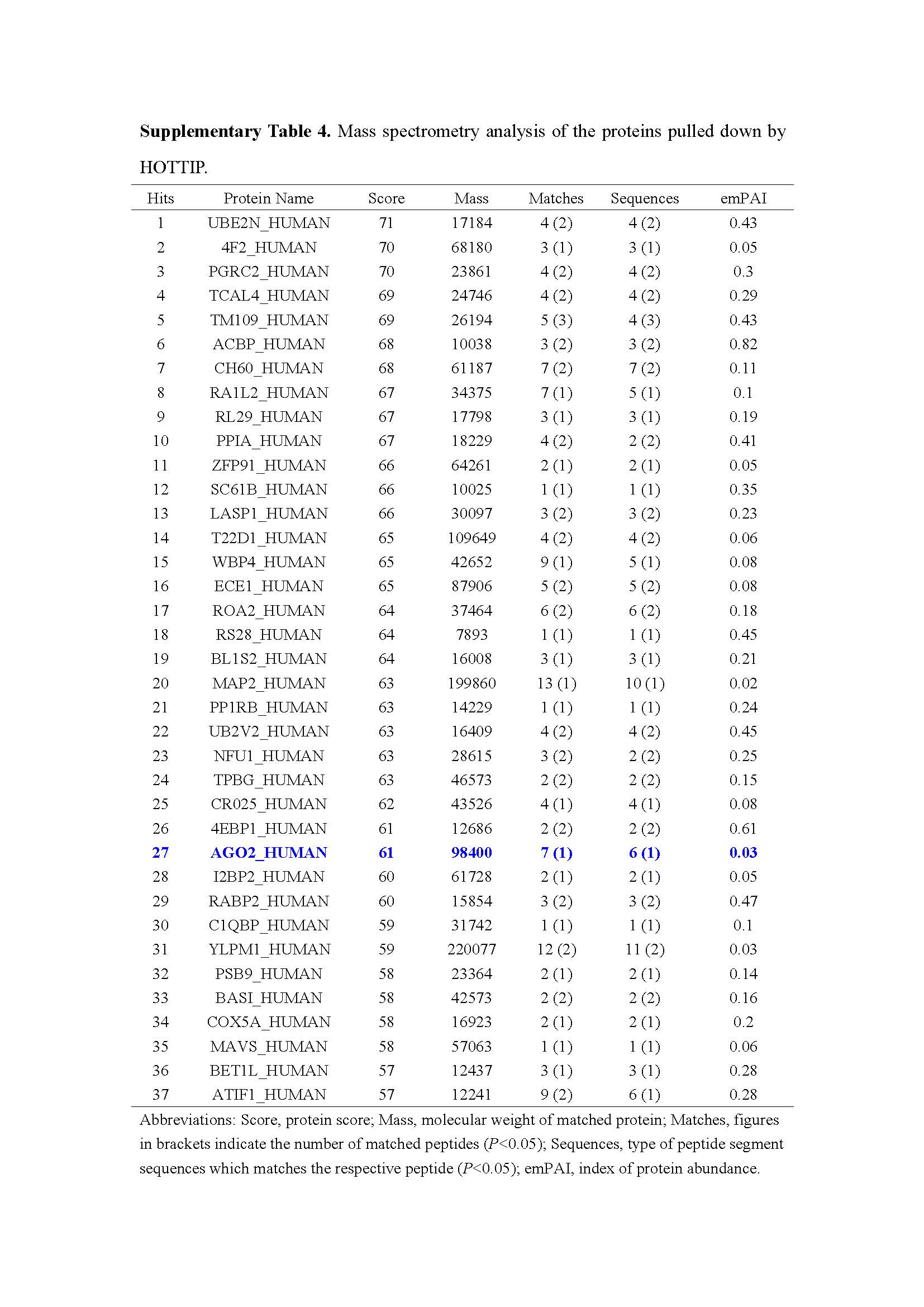

Supplement: Supplementary file 4 — Supplementary Table 4 [file 41419_2017_113_MOESM4_ESM.jpg]

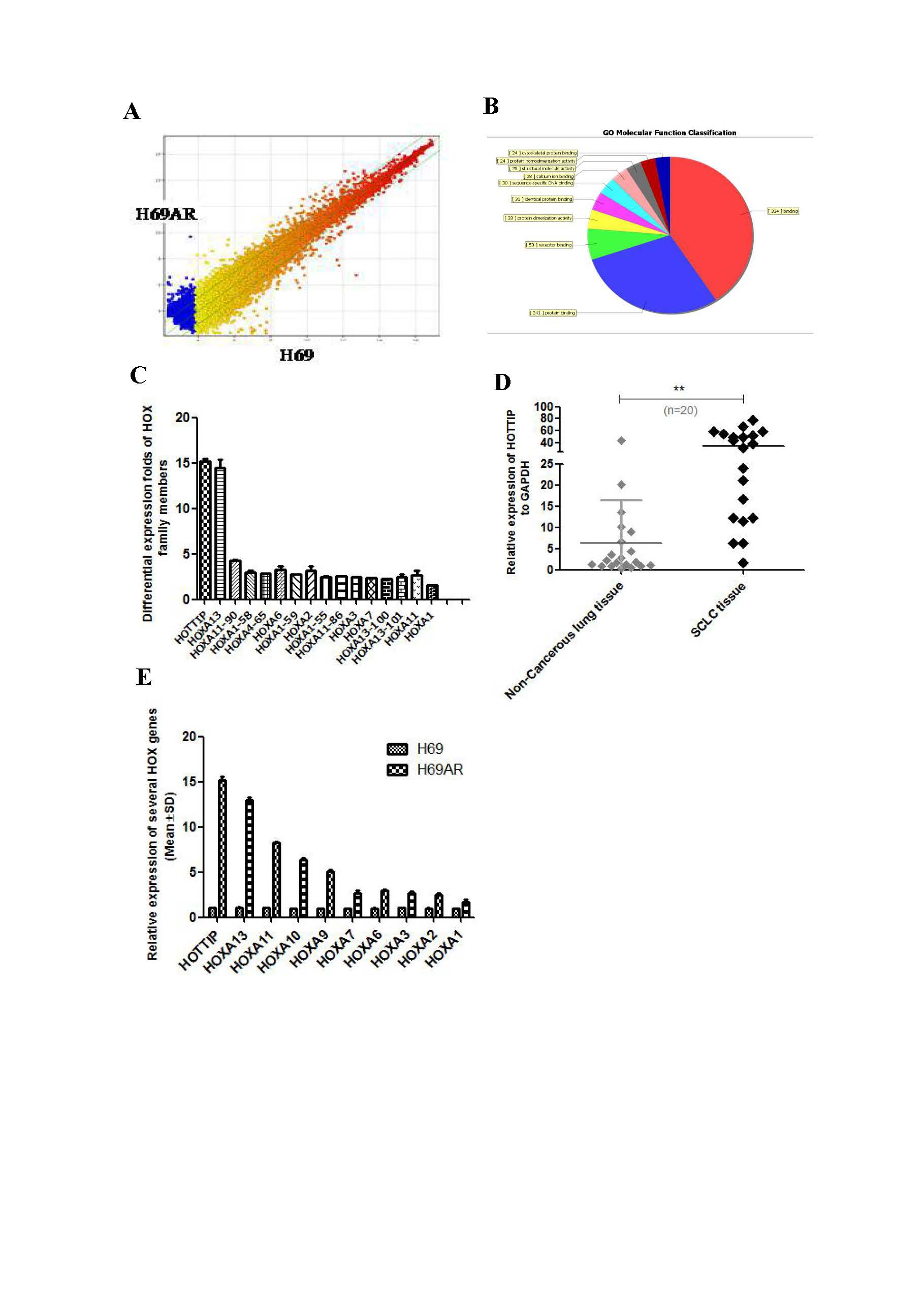

Supplement: Supplementary file 6 — Supplementary Figure 1 [file 41419_2017_113_MOESM6_ESM.jpg]

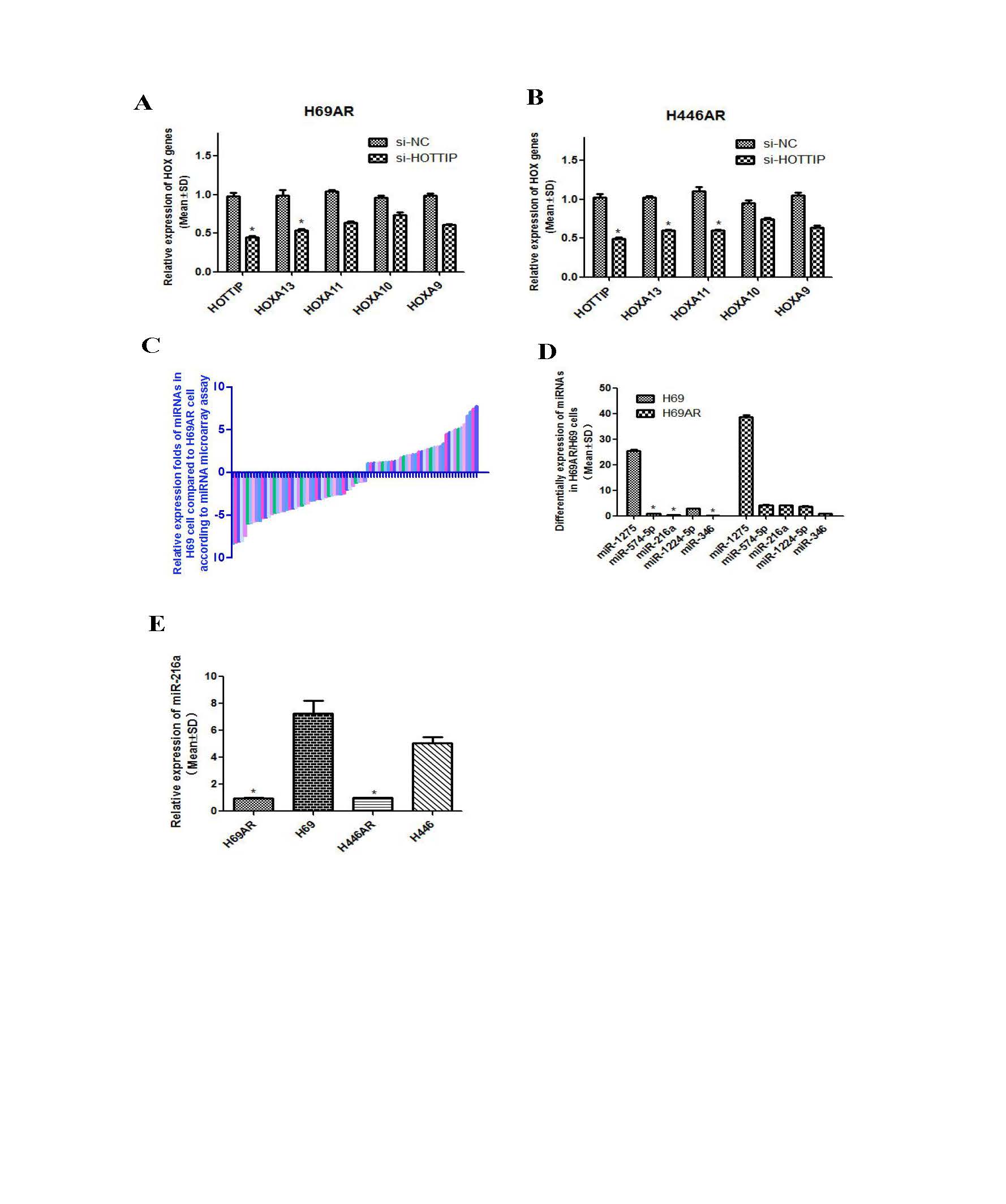

Supplement: Supplementary file 7 — Supplementary Figure 2 [file 41419_2017_113_MOESM7_ESM.jpg]
