# Supplementary material for: Long non-coding RNA HOTTIP promotes BCL-2 expression and induces chemoresistance in small cell lung cancer by sponging miR-216a
Source: Cell Death Dis. 2018 Jan 24;9(2):85. doi: 10.1038/s41419-017-0113-5 (PMC5833383; doi:10.1038/s41419-017-0113-5)
Supplement: Supplementary file 5 — Supplementary information [file 41419_2017_113_MOESM5_ESM.pdf]

Supplementary Table legends

Table 1. Differentially expressed genes in HOX gene family.

Table 2. Association of HOXA13 expression and clinical parameters.

Table 3. Differentially expressed miRNAs in H69/H69AR cells.

Table 4. Mass spectrometry analysis of the proteins pulled down by HOTTIP.

Primers:

HOTTIP Foward Primer(5'-3'): CCTAAAGCCACGCTTCTTTG

HOTTIP Reverse Primer(5'-3'): TGCAGGCTGGAGATCCTACT

HOXA13 Foward Primer(5'-3'): CTGCCCTATGGCTACTTCGG

HOXA13 Reverse Primer(5'-3'): CCGGCGGTATCCATGTACT

BCL-2 Foward Primer(5'-3'): GAACTGGGGGAGGATTGTGG

BCL-2 Reverse Primer(5'-3'): CCGTACAGTTCCACAAAGGC

miR-216a RT Primer(5'-3'): CTCAACTGGTGTCGTGGA

miR-216a Forward Primer(5'-3'): TGTCGCAAATCTCTGCAGG

miR-216a Reverse Primer(5'-3'): CAGAGCAGGGTCCGAGGTA

HOXA11 Foward Primer(5'-3'): TGCCAAGTTGTACTTACTACGTC

HOXA11 Reverse Primer(5'-3'): GTTGGAGGAGTAGGAGTATGTCA

HOXA10 Foward Primer(5'-3'): CTCGCCCATAGACCTGTGG

HOXA10 Reverse Primer(5'-3'): GTTCTGCGCGAAAGAGCAC

HOXA9 Foward Primer(5'-3'): TACGTGGACTCGTTCCTGCT

HOXA9 Reverse Primer(5'-3'): CGTCGCCTTGGACTGGAAG

HOXA7 Foward Primer(5’-3’): GCATAAGGACGAAGGTCCGA

HOXA7 Reverse Primer(5’-3’): AGACGCTTTTCCGACTGTCC

HOXA6 Foward Primer(5’-3’): TCCCGGACAAGACGTACAC

HOXA6 Reverse Primer(5’-3’): CGCCACTGAGGTCCTTATCA

HOXA3 Foward Primer(5’-3’): ATGCAAAAAGCGACCTACTACG

HOXA3 Reverse Primer(5’-3’): TACGGCTGCTGATTGGCATTA

HOXA2 Foward Primer(5’-3’): CCCCTGTCGCTGATACATTTC

HOXA2 Reverse Primer(5’-3’): TGGTCTGCTCAAAAGGAGGAG

HOXA1 Foward Primer(5’-3’): TCCTGGAATACCCCATACTTAGC

HOXA1 Reverse Primer(5’-3’): GCACGACTGGAAAGTTGTAATCC

GAPDH Forward Primers(5’-3’): GGGCTGCTTTTAACTCTG

GAPDH Reverse Primers(5’-3’): TGGCAGGTTTTTCTAGACGG

siRNA and shRNA sequences：

si-h-HOTTIP-1:

(positive-sense, 5'-3'): GCUGCUUUAGAGCCACAUA dTdT

(negative-sense, 3'-5'): dTdT CGACGAAAUCUCGGUGUAU

si-h-HOTTIP-2:

(positive-sense, 5'-3'): CCAGCUGCGAAUUCUUAAU dTdT

(negative-sense, 3’-5’):dTdT GGUCGACGCUUAAGAAUUA

si-h-HOTTIP-3:

(positive-sense, 5'-3'):CCUUGAUAUGCACGCAUAU dTdT

(negative-sense, 3’-5’): dTdT GGAACUAUACGUGCGUAUA

HOTTIP lentivirus vector (si-h-HOTTIP-1 was packaged by LV3 lentivirus vector):

Vector type: LV3（H1/GFP&Puro） sequence: 5’ GCUGCUUUAGAGCCACAUA dTdT 3’

si-HOXA13-1(sense, 5'-3'): 5′-AAUGUAUUUGUGCACCU GCUdTdT-′3

si-HOXA13-2(sense, 5'-3'): 5′/5fam/-CCG UCAUGUUUCUCUCUACGAdTdT-3′

miRNA mimics and inhibitor sequences：

HmiR-216a-FO：TGTCGCAAATCTCTGCAGG

HmiR-RE-6：CAGAGCAGGGTCCGAGGTA

Sequencing results of pcDNA3.1-HOTTIP expression:

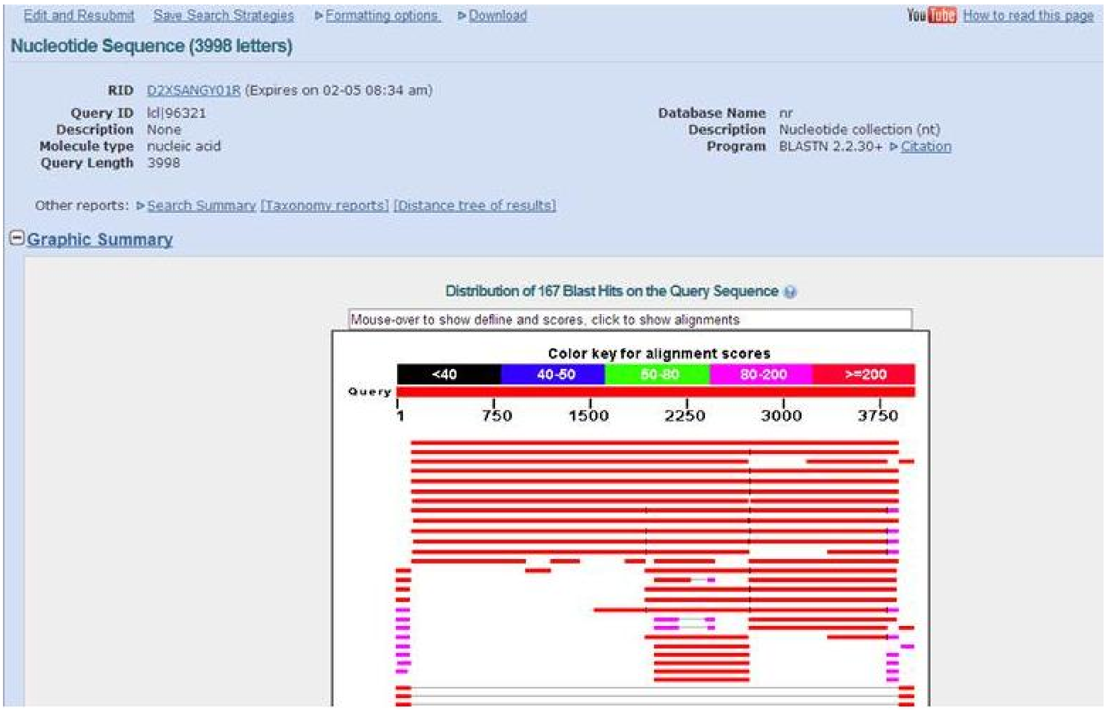

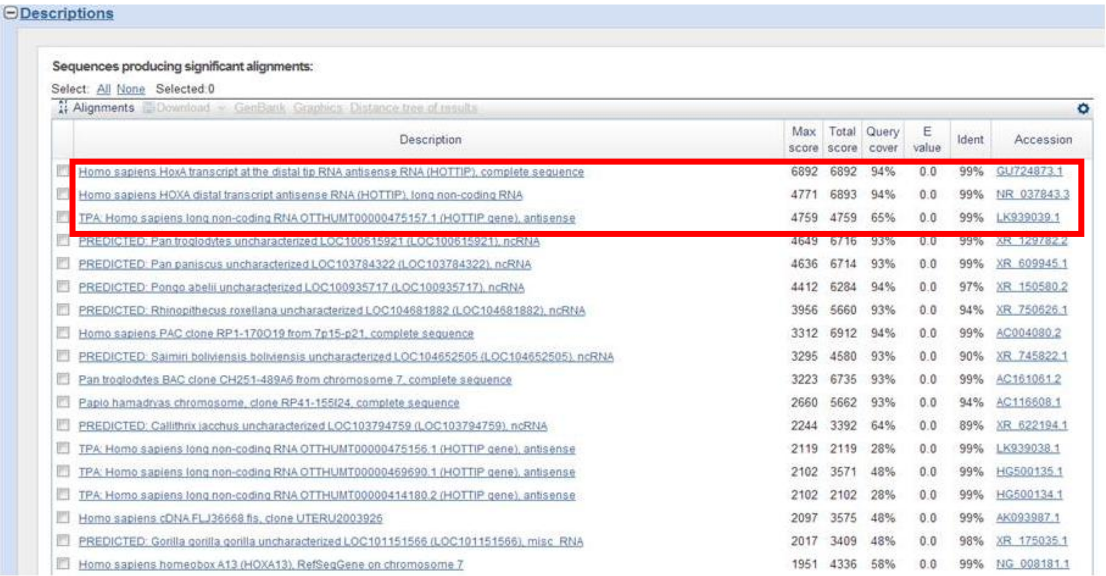

Descriptions

Sequences producing significant alignments:

Select: All None Selected:0

Alignments Download GenBank Graphics Distance tree of results

Description	Max score	Total score	Query cover	E value	Ident	Accession
Homo sapiens HoxA transcript at the distal tip RNA antisense RNA (HOTTIP), complete sequence	6892	6892	94%	0.0	99%	GU724873.1
Homo sapiens HOXA distal transcript antisense RNA (HOTTIP), long non-coding RNA	4771	6893	94%	0.0	99%	NR_037843.3
TPA: Homo sapiens long non-coding RNA OTTHUMT00000475157.1 (HOTTIP gene), antisense	4759	4759	65%	0.0	99%	LK939039.1
PREDICTED: Pan troglodytes uncharacterized LOC100615921 (LOC100615921), ncRNA	4649	6716	93%	0.0	99%	XR_129782.2
PREDICTED: Pan paniscus uncharacterized LOC103784322 (LOC103784322), ncRNA	4636	6714	93%	0.0	99%	XR_609945.1
PREDICTED: Pongo abelii uncharacterized LOC100935717 (LOC100935717), ncRNA	4412	6284	94%	0.0	97%	XR_150580.2
PREDICTED: Rhinopithecus roxellana uncharacterized LOC104681882 (LOC104681882), ncRNA	3956	5660	93%	0.0	94%	XR_750626.1
Homo sapiens PAC clone RP1-170O19 from 7p15-p21, complete sequence	3312	6912	94%	0.0	99%	AC004080.2
PREDICTED: Saimiri boliviensis boliviensis uncharacterized LOC104652505 (LOC104652505), ncRNA	3295	4580	93%	0.0	90%	XR_745822.1
Pan troglodytes BAC clone CH251-489A6 from chromosome 7, complete sequence	3223	6735	93%	0.0	99%	AC161061.2
Papio hamadryas chromosome, clone RP41-155I24, complete sequence	2660	5662	93%	0.0	94%	AC116608.1
PREDICTED: Callithrix jacchus uncharacterized LOC103794759 (LOC103794759), ncRNA	2244	3392	64%	0.0	89%	XR_622194.1
TPA: Homo sapiens long non-coding RNA OTTHUMT00000475156.1 (HOTTIP gene), antisense	2119	2119	28%	0.0	99%	LK939038.1
TPA: Homo sapiens long non-coding RNA OTTHUMT00000469690.1 (HOTTIP gene), antisense	2102	3571	48%	0.0	99%	HG500135.1
TPA: Homo sapiens long non-coding RNA OTTHUMT00000414180.2 (HOTTIP gene), antisense	2102	2102	28%	0.0	99%	HG500134.1
Homo sapiens cDNA FLJ36668 fis, clone UTERU2003926	2097	3575	48%	0.0	99%	AK093987.1
PREDICTED: Gorilla gorilla gorilla uncharacterized LOC101151566 (LOC101151566), misc_RNA	2017	3409	48%	0.0	98%	XR_175035.1
Homo sapiens homeobox A13 (HOXA13), RefSeqGene on chromosome 7	1951	4336	58%	0.0	99%	NG_008181.1

Plasmids sequencing map:

PSICHECK2.0- H –Bcl2–WT:

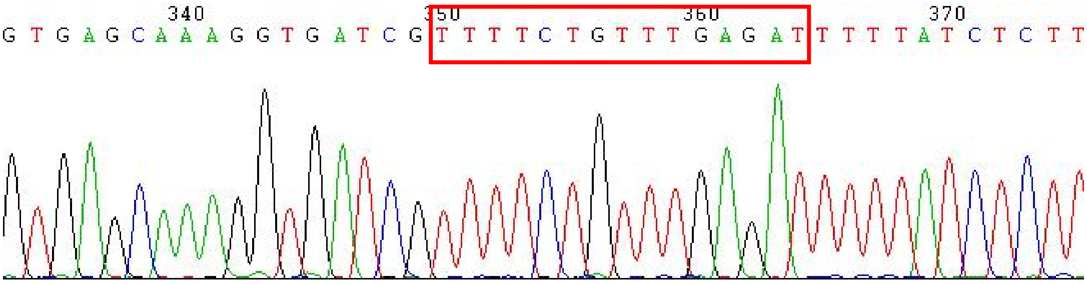

PSICHECK2.0- H – Bcl2 –Mut:

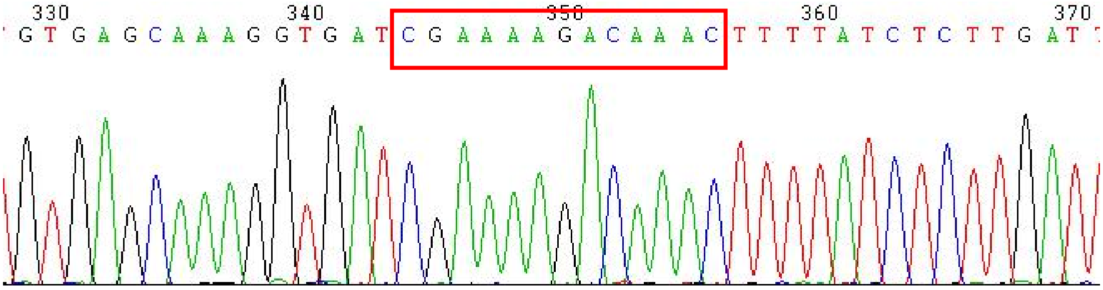

PSICHECK2.0- H –hottip–WT:

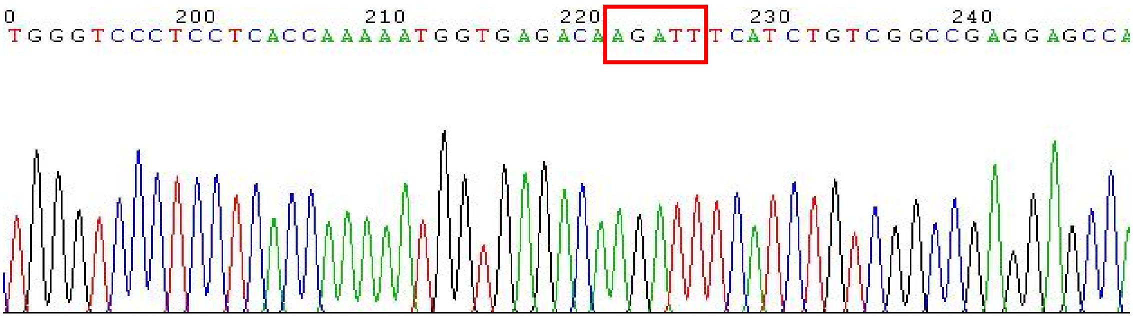

PSICHECK2.0- H –hottip–Mut:

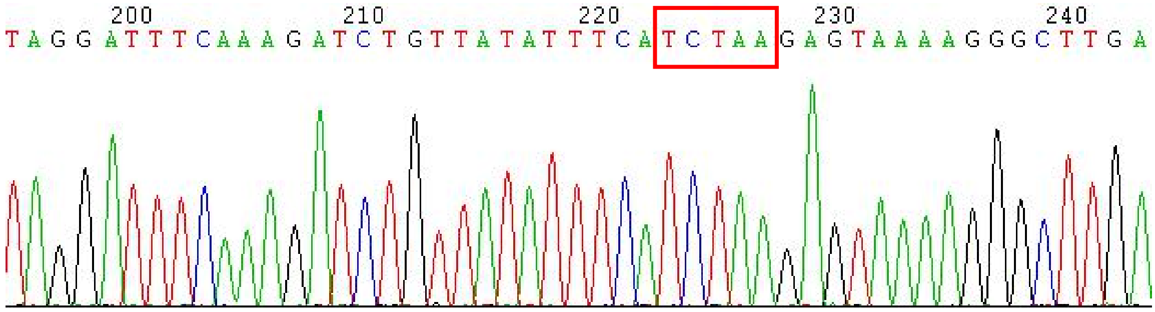

Antibodies information:

Rabbit anti-human HOXA13 monoclonal antibody (Abcam, ab175383)

Rabbit anti-human monoclonal antibody Bcl-2 (Santa Cruz Biotechnology, #5284)
